# Synergism of Plant Compound With Traditional Antimicrobials Against *Streptococcus* spp. Isolated From Bovine Mastitis

**DOI:** 10.3389/fmicb.2018.01203

**Published:** 2018-06-06

**Authors:** Natasha L. Maia, Mariana de Barros, Leandro L. de Oliveira, Silvia A. Cardoso, Marcelo H. dos Santos, Fabio A. Pieri, Teodorico C. Ramalho, Elaine F. F. da Cunha, Maria A. S. Moreira

**Affiliations:** ^1^Bacterial Disease Laboratory, Department of Veterinary, Universidade Federal de Viçosa, Viçosa, Brazil; ^2^Immunochemistry and Glycobiology Laboratory, Department of General Biology, Universidade Federal de Viçosa, Viçosa, Brazil; ^3^Department of Medicine and Nursing, Universidade Federal de Viçosa, Viçosa, Brazil; ^4^Synthesis of Agrochemicals Laboratory, Department of Chemistry, Universidade Federal de Viçosa, Viçosa, Brazil; ^5^Department of Basic Life Sciences, Universidade Federal de Juiz de Fora, Governador Valadares, Brazil; ^6^Department of Chemistry, Universidade Federal de Lavras, Lavras, Brazil

**Keywords:** guttiferone-A, 7-epiclusianone, *Streptococcus* spp., resistance, bovine, mastitis

## Abstract

Mastitis is an inflammation of the mammary gland that causes major losses in the dairy industry. *Streptococcus* spp. are among the main agents of this disease. Increased resistance to antibiotics is one of the causes of therapeutic failure. Plants, due to their broad chemodiversity, are an interesting source of new molecules with antibacterial activity. Using these compounds along with traditional antibiotics is a possible method for reversing resistance. The objective of this work was to determine the interactions between the activities of guttiferone-A and 7-epiclusianone, two active substances isolated from the fruits of *Garcinia brasiliensis*, and traditional antibiotics against *Streptococcus* spp. isolated from bovine mastitis and known to be resistant to them. First, the MIC for the antibiotics and bioactive compounds was determined, followed by their activities, alone and in combination. Then, their cytotoxicity was measured in bovine mammary epithelial cells. Finally, molecular docking simulations were performed to elucidate molecular details of the interactions between β-lactamase and the compounds binding to it (clavulanic acid, ampicillin, 7-epiclusianone, and guttiferone-A). The bacterial isolates were resistant to ampicillin and gentamicin. Both antibiotics showed predominantly synergistic antibacterial activities in combination with guttiferone-A or 7-epiclusianone. These two active substances were not cytotoxic at synergistic concentrations and both showed strong binding to β-lactamase, which may explain the reversal of ampicillin resistance. These substances are promising for the treatment of bovine mastitis.

## Introduction

Mastitis is the development of an inflammatory process in the mammary glands. It is a multifactorial disease and is the major cause of losses in the dairy industry (Vliegher et al., 2012). Among the main types of damage caused are the early slaughter of cows due to mammary parenchyma and decreased milk production, the commercial devaluation of animals, and the need for microbiological diagnosis, medication and veterinarian care (Halasa et al., 2007). Further damage is caused by milk discard, decreased milk quality as a result of microbiological, physical-chemical and sensorial changes and increased costs associated with those changes (Le Maréchal et al., 2011).

*Streptococcus* spp. are considered of importance in the etiology of mastitis in ruminants (Innings et al., 2005). *S. agalactiae* is one of the most commonly isolated infectious agents in milk samples from bovines affected by mastitis (Alemu et al., 2014). *S. uberis* has great importance in the etiology of clinical episodes of environmental mastitis worldwide, especially when the contamination of the animal occurs through environmental sources and organic matter (Lopez-Benavides et al., 2007).

Antimicrobial therapy is the most commonly used method of mastitis control (Fernandes, 2006). However, there has been an increase in bacterial resistance to traditional antimicrobial agents and consequently an increase in therapeutic failures, as well as increased treatment costs. The antibiotic resistance mechanisms are categorize according to the biochemical route involved in resistance, as follows: (i) modifications of the antimicrobial molecule, (ii) prevention to reach the antibiotic target (by decreasing penetration or actively extruding the antimicrobial compound), (iii) changes and/or bypass of target sites, and (iv) resistance due to global cell adaptive processes (Munita and Arias, 2016). In this context, natural products have been an important source for the discovery of new therapeutic agents, due to their broad chemodiversity (Ionta et al., 2015).

**FIGURE 1:**
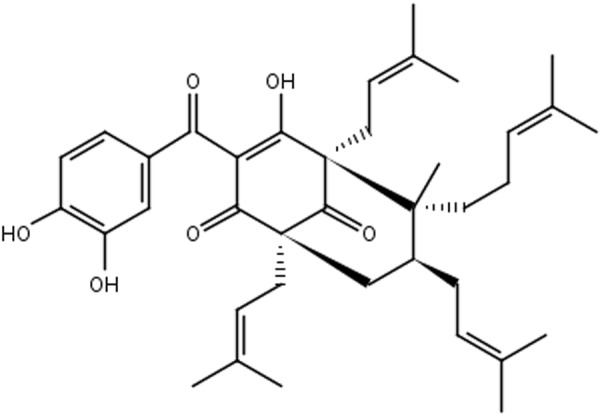
Chemical structure of guttiferone-A.

**FIGURE 2:**
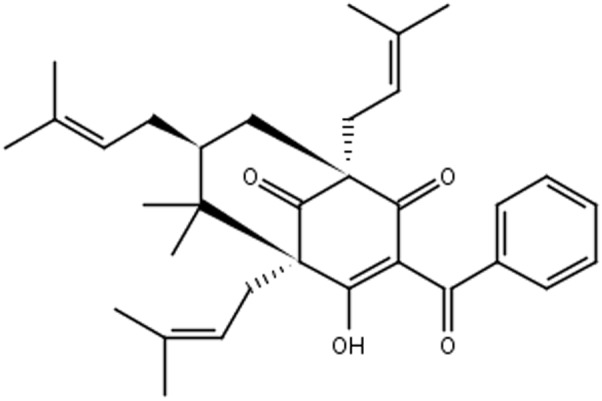
Chemical structure of 7-epiclusianone.

Plants of *Garcinia* spp. are rich sources of bioactive compounds (Almeida et al., 2008). *Garcinia brasiliensis* is a tree species native to the Amazon region, and cultivated throughout the Brazilian territory, popularly known as “bacupari mirim.” Guttiferone-A (Gut-A) (**Figure 1**) is one of the most abundant bioactive substances isolated from its fruits, and there are several reports of its pharmacological properties: antimicrobial actions against *Staphylococcus aureus* and *Bacillus cereus* (Dias et al., 2012), and anti-HIV, trypanocidal, antispasmodic, antioxidant, antitumor and protease inhibitor activities (Gustafson and Mckee, 1992; Ngouela et al., 2006; Coelho et al., 2008; Martins F.T. et al., 2009; Martins M.D. et al., 2009). 7-epiclusianone (7-epi) (**Figure 2**) derived from gut-A exhibits a broad spectrum of biological activities (Ionta et al., 2015), including toxicity to *Trypanosoma cruzi* trypomastigotes (Alves et al., 1999), anti-HIV (Piccinelli et al., 2005), vasodilation (Cruz et al., 2006), anti-anaphylaxis (Neves et al., 2007), antinociception and anti-inflammatory activities (Santa-Cecília et al., 2011), antimicrobial activity against *Streptococcus mutans* (Almeida et al., 2008) and other *Streptococcus* spp. (Barros et al., 2017), inhibition of cell reproduction in MDCK and IIN-5 cells (Vieira et al., 2009), and antiproliferation activity against cancer cells (Almeida et al., 2008).

The synergistic potential for these two bioactive substances with traditional antimicrobials is not known and may become a promising treatment for bovine mastitis, since the number of bacterial strains that are resistant to these agents is increasing. There is, therefore, a need for studies concerning the action spectrum, the dosage, the cell viability after treatment, among other pharmacological aspects of these compounds.

The aim of this work was to determine the interaction between gut-A or 7-epi and traditional antimicrobials in their activity against *Streptococcus* spp. isolates from mastitis known to be resistant to traditional antimicrobials.

## Materials and Methods

### Bacterial Isolates

Isolates of the genus *Streptococcus* representing etiological agents of contagious and environmental mastitis were kindly provided by the Brazilian Agricultural Research Corporation (EMBRAPA), Gado de Leite (Juiz de Fora, MG, Brazil).

From the -80°C stocks, cultures were reactivated in Tryptic Soy Broth (TSB, Sigma-Aldrich, St. Louis, MO, United States) at 37°C until they reached a density of 0.5 on the McFarland scale corresponding to 1.0 × 10^8^ Colony Forming Units (CFU)/ml of *Streptococcus* spp. culture (optical density 0.1 at 550 nm, UV-Vis BioMate 3S, Thermo Science^®^). Subsequently, the bacterial isolates were diluted to a concentration of 1.0 × 10^7^ CFU/mL for use in tests.

### Bioactive Substances and Traditional Antibiotics

The bioactive substances gut-A and 7-epi were studied. These substances were obtained from fruits of *Garcinia brasiliensis* (Martins F.T. et al., 2009), cultivated in the herbarium of the Universidade Federal de Viçosa (Viçosa, MG, Brazil), being the voucher specimen of the species, deposited under the number VIC2604.

The working solutions were prepared by the addition of 500 μL of dimethyl sulfoxide (DMSO, VETEC, Rio de Janeiro, Brazil) to 1 mg of each lyophilized substance, followed by the addition of 500 μL of phosphate buffered saline (PBS) to a final concentration of 1 mg/mL in 50% DMSO.

The following antibiotics used in the treatment of bovine mastitis were tested: ampicillin (AMP), ceftriaxone (CRO), amoxicillin with clavulanate (AMC), and gentamicin (GEN). Working solutions were prepared at a final concentration of 1 mg/mL in PBS.

Bioactive substances and antibiotics were tested on both isolates in triplicate and with four replicates. The working solutions were prepared on the day of the experiment in order to avoid possible degradation.

### Determination of Minimum Inhibitory Concentration – MIC

The broth microdilution technique was used, according to the CLSI (2012). To obtain the different concentrations of the antibiotics and bioactive substances a serial dilution of 1:2 was carried out from the working solutions. 100 μL of each dilution and 100 μL of the inoculum of each bacterial isolate were added to the wells of a 96-well microtiter plate. For the control multiplication, 100 μL of inoculum was used together with 100 μL of TSB and for the sterile control, 200 μL of TSB was added to the wells. After incubation for 24 h at 37°C, the optical densities of the cultures in the plates were read in a spectrophotometer. The MIC was considered as the lowest concentration capable of preventing bacterial multiplication, compared with the controls. The MIC values were obtained by calculating the means of the results.

### Interactions Between Antibiotics and Bioactive Substances

To determination interactions between antibiotics and bioactive substances a synergism test was applied by applying the Checkerboard method with adaptations (Reuk-ngam et al., 2014). Antibiotics that presented MIC values above or equal to the cut-off points corresponding to *Streptococcus* spp. resistance were used.

From the working solutions of antibiotics and bioactive substances the concentrations were readjusted to 2 × ; 1 × ; 0.5 × ; 0.25 × and 0.125 × the MIC (final concentration in the well).

The wells of a microtiter plate were filled with 50 μL of different concentrations of the antibiotic solution (ordinate), 50 μL of the different concentrations of the solution of the bioactive substance (abscissa), and 100 μL of the bacterial inoculum. For the control for each substance, 50 μL of PBS, 50 μL of antibiotic solution or bioactive substance and 100 μL of bacterial inoculum were added to the wells. After incubation at 37°C for 24 h, the plates were read in a spectrophotometer at 595 nm.

The synergism tests were performed in triplicate, using the MIC/FIC checkerboard proposed by Fratini et al. (2017) (Synergy when FICI < 1; Commutative effect when FICI value = 1; Indifferent effect when 1 < FICI ≤ 2 and Antagonistic when FICI > 2), the isobolograms were prepared according to Hewlett (1969).

### Cytotoxicity Test

The cytotoxicity of the bioactive substances was evaluated *in vitro* in bovine mammary gland epithelial cells (MAC-T) using the MTT reduction method (3- [4,5-dimethyl-thiazol-2-yl] -2,5 -diphenyl tetrazolium bromide), according to Mosmann (1983).

MAC-T cells were cultured in 96-well microtiter plates (Corning Incorporated – Life Sciences, NY, United States) in Dulbecco’s Modified Eagle Media (DMEM, Gibco-BRL, Grand Island, NY, United States) supplemented with 10% FBS (Cultilab, Campinas, SP, Brazil), penicillin (100 μg/mL) and streptomycin (100 μg/mL). The plate wells received the concentration of 2 × 10^4^ cells/ml. The flasks were incubated at 37°C in a humid atmosphere with 5% CO_2_ for 16 h to obtain a monolayer with 90–95% confluence as visualized using an inverted microscope (Olympus IX70, Olympus Corporation, Shinjuku, Tokyo, Japan).

The antibiotic and bioactive substance solutions were prepared based on their MIC values, using the same concentrations as in the synergism tests. The wells were filled with 100 μL of antibiotic solution and 100 μL of DMEM. For negative controls only 100 μL of DMEM medium was used. The microplates were reincubated for 72 h, and thereafter the MTT assay was performed.

The supernatant was discarded and 50 μL of 1 mg/mL MTT in PBS was added to each well, the plates were then returned to the previous incubation conditions for an additional 4 h. The media was discarded again and 100 μL of DMSO was added to each well. After incubation for 1 h with agitation at 37°C, the O.D._550__nm_ was measured in a spectrophotometer and the results were analyzed. The tests were performed in quadruplicate with two replicates.

### Molecular Docking

To study protein-binder interactions the molecular docking method was used. This method is based on the key-lock model and the study is performed via simulation of rotational and translational movements of the ligand at a specific site, usually the active site of the protein (Gonçalves, 2008).

In this work, the calculations were performed in the program Molegro Virtual Docker (MVD) (Thomsen and Christensen, 2006). The structural data for the β-lactamase enzyme were obtained from the Protein Data Bank (PDB), code 2Y9 (Berenbaum, 1989), and the interaction energies between this protein and the ligands clavulanic acid, AMP, 7-epi and gut-A were calculated.

## Results

### Minimal Inhibitory Concentration – MIC

The MIC values are shown in **Table 1**. *S. agalactiae* and *S. uberis* were resistant to AMP and GEN. *S. uberis* were resistant to CRO too. gut-A and 7-epi showed good results for these two bacteria, with low MIC values. To the others tested drugs, the isolates presented sensitivity profiles.

**Table 1:** MIC results for *Streptococcus agalactiae* and *Streptococcus uberis* isolates.

	MIC (μg/mL)	
	*S. agalactiae*	*S. uberis*
Gut-A	7.81	15.62
7-epi	0.98	3.90
AMP	31.25^1^	125.0^1^
GEN	7.81^1^	15.62^1^
CRO	<0.49^2^	>250.0^1^
AMC	<0.49^2^	<0.49^2^

*^1^Resistant. ^2^Sensitive to all tested concentrations. Sensitive interpretive criteria to Streptococcus spp: AMP ≤ 0.25; GEN ≤ 0.4; CRO ≤ 0.5 and AMC ≤ 0.12. Gut-A: Guttiferone-A, 7-epi: 7-epiclusianone, AMP: ampicillin, GEN: gentamicin, CRO: ceftriaxone, AMC: amoxicillin with clavulanic acid.*

### Association Test

**FIGURE 3:**
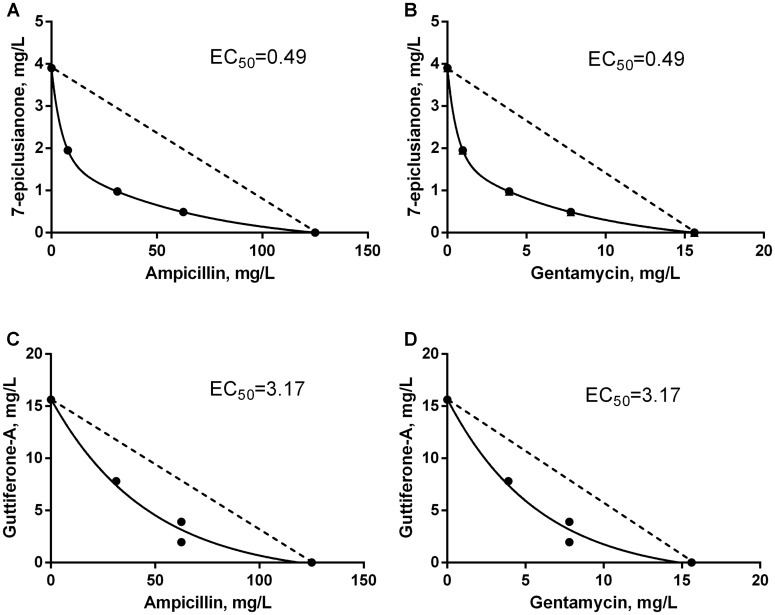
Isobolograms of the synergistic interaction between antimicrobials and bioactive substances for *Streptococcus uberis*. EC_50_ = effective concentration of bioactive compound that leading to the twofold reduction of antibiotic’s MIC. Dotted line FICI = 1. **(A)** Synergistic interaction between 7-EPI and AMP. **(B)** Synergistic interaction between 7-EPI and GEN. **(C)** Synergistic interaction between GUT-A and AMP. **(D)** Synergistic interaction between GUT-A and GEN.

**FIGURE 4:**
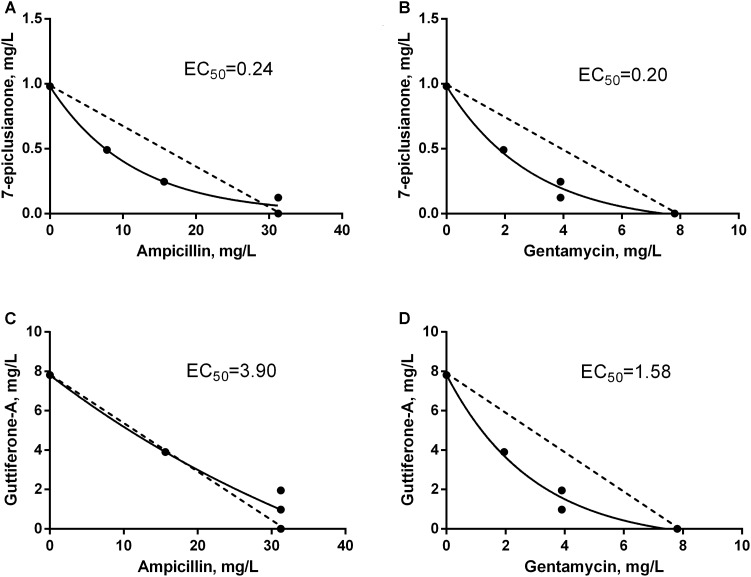
Isobolograms of the interaction between antimicrobials and bioactive substances for *Streptococcus agalactiae*. EC_50_ = effective concentrations that leading to the twofold reduction of antibiotic’s MIC. Dotted line FICI = 1. **(A)** Synergistic interaction between 7-EPI and AMP. **(B)** Synergistic interaction between 7-EPI and GEN. **(C)** Additive interaction between GUT-A and AMP. **(D)** Synergistic interaction between GUT-A and GEN.

The synergism of 7-epi or gut-A in combination with AMP or GEN on the bacterial growth was analyzed by the checkerboard assay, resulting in a total of eight combinations of traditional antimicrobials with bioactive substances. Among these combinations, seven synergistic interactions were observed and only one additive interaction (**Figures 3**, **4** and **Table 2**).

**Table 2:** MIC and EC50 of biocomposite that reducing twice the MIC of the appropriate antibiotic and FICI values of those antibiotics combined with 7-epi or gut-A.

	Combination	MIC^a^	EC_50_^a^	FICI
*S. uberis*	AMP × 7-epi	62.50	0.49	0.63
	GEN × 7-epi	7.81	0.49	0.63
	AMP × gut-A	62.50	3.17	0.70
	GEN × gut-A	7.81	3.17	0.70
*S. agalactiae*	AMP × 7-epi	15.63	0.24	0.74
	GEN × 7-epi	3.91	0.20	0.70
	AMP × gut-A	15.63	3.90	1.00
	GEN × gut-A	3.91	1.58	0.70

*^a^The concentrations are given in μg/mL.*

### Cytotoxicity Test

**FIGURE 5:**
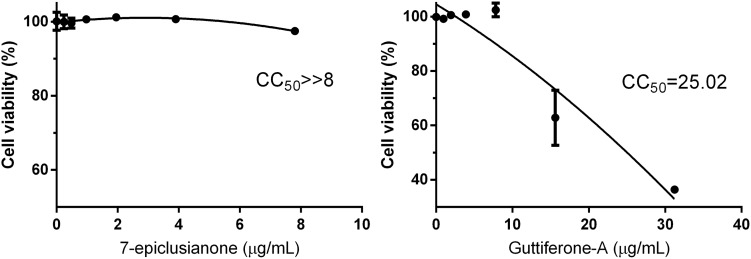
Cell viability of bovine mammary gland epithelial cells (MAC-T) in response to different concentrations for each biocomposite. CC_50_ as the cytotoxic concentration of the biocomposite to cause death to 50% of viable cells.

Quantitative analysis of the absorbance values obtained (**Figure 5**) showed that 7-epi did not cause cytotoxic effects in MAC-T cells at any of the tested concentrations. This substance has been shown to be safe for use up to 2 × MIC, without changes in the viability of MAC-T cells.

On the other hand, gut-A showed cytotoxic effects on MAC-T cells at the MIC. Although gut-A causes from 23.60 to 56.1% toxicity in MAC-T cells, these changes only occurred at concentrations greater than 0.5 × MIC. As the concentration used in the synergism test was ≤ 0.5 × MIC, this bioactive substance can be used in therapeutic protocols in combination with traditional antimicrobials.

Considering the possibility of using bioactive substances as potentiators of the effects of synthetic antimicrobials rendered inefficient due to bacterial resistance, the results can be said to be promising and encouraging, and should stimulate further studies.

### Molecular Docking

To investigate the molecular details of the intermolecular interactions among AMP, clavulanic acid, 7-epi, gut-A and β-lactamase, docking studies were performed. The results of the energy calculations are shown in **Table 3**.

**Table 3:** Energy values calculated during docking between the ligands and β-lactamase, using the MVD software (Thomsen and Christensen, 2006).

	Compounds			
Type of energy	7-epiclusianone	Gutiferone-A	Clavulanic acid	Ampicillin
MolDock Score (kcal mol^-1^)	-114.04	-138.72	-61.33	-86.71
Intermolecular (kcal mol*^-^*^1^)	-134.89	-179.83	-68.13	-105.32
Hydrogen bonding (kcal mol*^-^*^1^)	-2.55	-3.48	-7.23	-3.75
Interaction with Ser70 (kcal mol*^-^*^1^)	-7.65	-10.78	-5.44	-6.31
Intramolecular (kcal mol*^-^*^1^)	20.85	41.11	6.80	18.60

Among the four inhibitors, the calculated total and intermolecular interaction energies with β-lactamase presented the highest stability for 7-epi and gut-A. This result corroborates the MIC data, where inhibition was more effective for these two compounds compared with AMP.

## Discussion

### MIC

The low MIC values of gut-A and 7-epi confirm the strong antibacterial potential of these bioactive substances. It is also worth mentioning that the isolates tested here are wild and subjected to field selective pressure; even so, gut-A and 7-epi were able to inhibit the multiplication of these bacteria.

Inhibition of *S. uberis* and *S. agalactiae* multiplication is novel and important data concerning the antimicrobial action of gut-A on field isolates from bovine mastitis. The importance of these substances for use as therapy in the control of mastitis caused by these pathogens is demonstrated for the first time in this study.

The bacteria tested showed resistance to AMP, in contrast to the reports by CLSI (2014) and others (Erskine et al., 2002; Rossitto et al., 2002; Pitkälä et al., 2008; Loures, 2011) who found *Streptococcus* spp. to be highly sensitive to the penicillin group. This is probably due to the selective pressure found in the environment where the isolates were obtained.

CRO was not able to control the multiplication of *S. uberis*, although *S. agalactiae* is fully sensitive. By other hand, *S. agalactiae* was resistant to GEN. However, the MIC of GEN obtained for *S. uberis* revealed a moderate or intermediate sensitivity condition, in agreement with the study by Kaspar (2006).

Clearly, the structuring of a cycle of bacterial resistance, fed by indiscriminate and incorrect use of traditional antimicrobials, is being observed. The MIC results for the antimicrobials used in the treatment of mastitis reveal a need for the use of higher doses than the therapeutic doses or exchange of therapeutic drugs to achieve successful treatment. Bacterial resistance is a public health problem as well, as humans can ingest resistant bacteria in milk and dairy products, which can lead to complications in treatment of human diseases.

Combinations with other antimicrobials and/or the use of alternative drugs are options to decrease the process of selection of resistant bacteria.

### Association Test

There was a potentiation of the effects of AMP and GEN when they were associated with the bioactive substances. Berenbaum (1989) previously reported that the combination of antimicrobial agents of different origins can cause different effects than the same compounds used in isolation.

The synergistic interaction between AMP and 7-epi was predominant, however in the final third of the dispersion curve there was a change in the tendency of this response, and an indifferent effect was seen (**Figure 4A**). However, this behavior should not influence the use of this combination of drugs for the treatment of mastitis, since the concentrations where the synergism occurred were those selected for therapeutic treatment (below 0.8 × MIC for AMP and above 0.2 × MIC for 7-epi).

An additive effect was observed at low concentrations of AMP in combination with gut-A, however this trend was not maintained. As the concentrations of the two substances increased, an indifferent interaction occurred (**Figure 4C**). Despite the indifferent interaction at high concentrations, the additive effect initially observed between AMP and gut-A is a satisfactory result, since even at subinhibitory concentrations AMP exerted better antimicrobial activity in combination with gut-A compared with its individual activity.

According to Kumari et al. (2017), the plant extract having significant MIC will not necessarily shows synergistic effect with antibiotics.

The main mechanism of resistance to β-lactams of the AMP group is inactivation of the lactam ring by β-lactamases. Thus, the synergism found between AMP and 7-epi, and AMP and gut-A could be explained by the inhibitory binding of these substances to β-lactamase, which would prevent the destruction of AMP, and thus reverse the resistance to this antimicrobial compound.

Other studies have also been conducted seeking the synergism between compounds or plant extracts and traditional antimicrobial order to help fight against microbial emerging drug resistance (Araújo et al., 2014; Biasi-Garbin et al., 2015; Moussaoui and Alaoui, 2016; Wikaningtyas and Sukandar, 2016; Sanhueza et al., 2017). But many of them work with human isolates and reference strains. There are still few works in the literature with the present focus described here, using field isolates that underwent selective pressure from the farm environment.

Therefore, these results suggest a successful path for the treatment of bovine mastitis, since the occurrence of populations of bacteria resistant to traditional antimicrobials is increasing. Thus, the use of combinations of drugs with different origins is promising in veterinary medicine.

### Molecular Docking

Currently, molecular modeling based methods involving docking studies and also molecular dynamics simulations are suitable tools to adjust ligands at target sites and to estimate interaction energy (affinity) (Sales et al., 2017; Shanmugam and Jeon, 2017). In fact, molecular docking is a well-established technique applied to numerous cases (Gioia et al., 2014; Honarparvar et al., 2014). This methodology explores the behavior of small molecules in the binding site of a target protein (Pagadala et al., 2017) and are being used to elucidation of enzymatic mechanisms or the depiction of the quaternary structure of biological protein complexes (de Ruyck et al., 2016). It is important to keep in mind that three primary factors are known to influence the conformation of a ligand bonded to a protein: hydrogen bonding, binding energies and hydrophobic-hydrophobic interactions. It is important to keep mind that it is possible to identify quantitatively the existence of hydrogen bonding and molecular interactions in the docked conformation with the Docking tools, which may lead to best fit of the ligand to the enzyme pocket.

**FIGURE 6:**
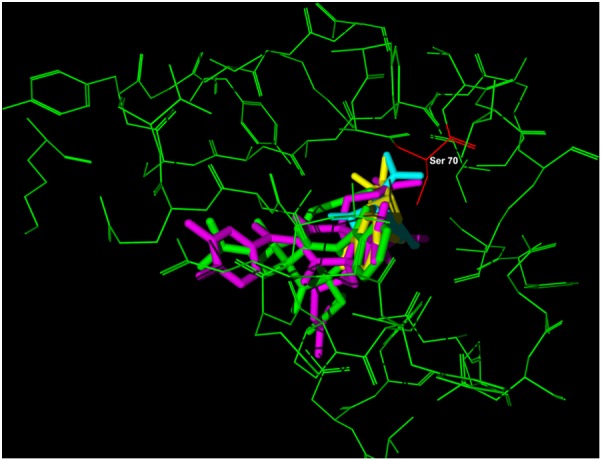
Representation of the overlap of the four inhibitors – clavulanic acid (blue), 7-epiclusianone (green), guttiferone-A (pink) and ampicillin (yellow) – at the beta-lactamase binding site (green macromolecule) amino acid Ser70 (red).

Since 7-epi and gut-A have a skeleton with a greater number of atoms than AMP, the intramolecular energy disfavors these compounds. The initial acylation of the Ser70 residue to form the acyl-lactamase complex is the first step of the reaction with clavulanic acid, a known β-lactamase inhibitor, so the interaction energy of this residue with the other ligands was verified and gut-A presented a more stable energy value than the other compounds. In **Figure 6**, the overlap of the three inhibitors at the β-lactamase binding site is shown and Ser70 is highlighted.

**FIGURE 7:**
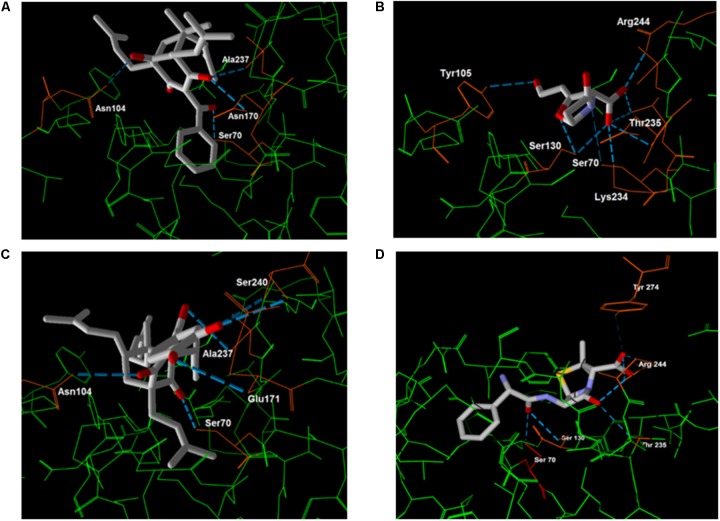
Molecular docking between β-lactamase and 7-epiclusianone **(A)**, clavulanic acid **(B)**, gutiferone **(C)** and ampicillin **(D)**. The hydrogen bonding interactions between the inhibitor and the residues (orange) are shown with a blue dash.

Regarding the hydrogen bonding, clavulanic acid presented a more stable energy value than the other compounds. In **Figure 7**, the amino acids responsible for such interactions with the inhibitors are identified.

The greater stability of binding of 7-epi and gut-A may also be related to the reversal of the resistance of *S. agalactiae* and *S. uberis*, since by binding more stably to β-lactamase they might act as inhibitors of the enzyme, preventing the destruction of ampicillin and allowing it to interfere with bacterial cell wall synthesis.

The molecular docking is an excellent tool and has been used for various purposes: to understand the mechanism of development of resistance to microorganisms and to obtain detailed information about the active site of the enzyme (Daga et al., 2010); to investigate antibacterial and adjuvant drug properties (de Araújo et al., 2016); to understand the mechanism of action of antimicrobial drugs (Jena et al., 2014), to understand mechanism of drug resistance and aid in designing potent inhibitors (Malathi and Ramaiah, 2016), to developing a new class of antimicrobial agents. (Elavarasan et al., 2014); to study of target proteins involved in antibacterial mechanisms of action (Alves et al., 2014) and others.

Synergisms of traditional antibiotics with compounds obtained from plants have many advantages such as increased efficiency, reduction of undesirable effects, increased stability or bioavailability of the free agents and obtaining a suitable therapeutic effect at lower doses (Aiyegoro and Okoh, 2009).

Therefore, these results open new perspectives for studies of resistance mechanisms, since the molecular details presented here provide clues that can form the basis for the design of new prototypes in the fight against resistance to ampicillin.

## Conclusion

The bioactive substances 7-epi and gut-A act in synergism with AMP and GEN at concentrations below the MIC. These substances were able to reverse the resistance of *S. agalactiae* and *S. uberis* to the antibiotics, proving to be promising compounds for the treatment of bovine mastitis.

## Author Contributions

All authors listed have made a substantial, direct and intellectual contribution to the work, and approved it for publication.

## Conflict of Interest Statement

The authors declare that the research was conducted in the absence of any commercial or financial relationships that could be construed as a potential conflict of interest.
